# Mercury enrichment indicates volcanic triggering of Valanginian environmental change

**DOI:** 10.1038/srep40808

**Published:** 2017-01-20

**Authors:** Guillaume Charbonnier, Chloé Morales, Stéphanie Duchamp-Alphonse, Stéphane Westermann, Thierry Adatte, Karl B. Föllmi

**Affiliations:** 1 Institute of Earth Sciences, Géopolis, University of Lausanne, CH-1015 Lausanne, Switzerland; 2 Marine Palynology Group, Institute of Earth Sciences, University of Utrecht, Heidelberglaan 24, 3584 CS Utrecht, The Netherlands; 3 Laboratoire GEOPS, Bâtiment 504, Université Paris Sud, UMR 8148, Orsay F91405, France; 4 Cantonal agency for environmental protection, Rue des Creusets 5, CH-1950 Sion, Switzerland

## Abstract

The Valanginian stage (Early Cretaceous) includes an episode of significant environmental changes, which are well defined by a positive δ^13^C excursion. This globally recorded excursion indicates important perturbations in the carbon cycle, which has tentatively been associated with a pulse in volcanic activity and the formation of the Paraná-Etendeka large igneous province (LIP). Uncertainties in existing age models preclude, however, its positive identification as a trigger of Valanginian environmental changes. Here we report that in Valanginian sediments recovered from a drill core in Wąwał (Polish Basin, Poland), and from outcrops in the Breggia Gorge (Lombardian Basin, southern Switzerland), and Orpierre and Angles (Vocontian Basin, SE France), intervals at or near the onset of the positive δ^13^C excursion are significantly enriched in mercury (Hg). The persistence of the Hg anomaly in Hg/TOC, Hg/phyllosilicate, and Hg/Fe ratios shows that organic-matter scavenging and/or adsorbtion onto clay minerals or hydrous iron oxides only played a limited role. Volcanic outgassing was most probably the primary source of the Hg enrichments, which demonstrate that an important magmatic pulse triggered the Valanginian environmental perturbations.

The Valanginian stage (Early Cretaceous, ~137–132 Ma) recorded an episode of pronounced palaeoenvironmental changes, which are marked by a globally recorded positive δ^13^C excursion in carbonate (1.5 to 2‰ amplitude), and in organic fractions (4 to 5‰ amplitude) of marine and terrestrial origin^1^,^2^,^3^,^4^,^5^,^6^. The Valanginian carbon-isotope excursion is also known as the “Weissert event or episode”^3^,^7^,^8^. Its onset near the early/late Valanginian boundary (*B. campylotoxus-S. verrucosum* ammonite Zones) coincides with a phase of warmer climate conditions associated with enhanced humidity^9^,^10^,^11^,^12^, major changes in the evolution of marine plankton, and the drowning of tropical and subtropical marine shallow-water carbonate ecosystems^2^,^13^,^14^,^15^,^16^.

Intense volcanic activity related to the emplacement of large igneous provinces (LIPs) is often considered as a trigger of past environmental changes^17^ and also in the case of the Valanginian Weissert episode, a link has been proposed with increased volcanic activity related to the continental Paraná-Etendeka LIP^2^,^3^,^14^,^18^,^19^. The onset of the perturbation is ascertained to 136 ± 1 Ma by U-Pb ages from tuff layers in California^20^, or more recently to 135.22 ± 1 Ma by a re-evaluation of an U-Pb age from tuff layers in the Neuquén Basin^21^, and an update of the Valanginian-Hauterivian astrochronological time scale^22^. The Valanginian Weissert episode may has therefore coincided with the onset of the eruptive phase of the Paraná-Etendeka, which has recently been dated between 134.6 ± 0.6 Ma and 134.3 ± 0.8 Ma^23^,^24^. However, the scarcity of direct radiometric ages and the uncertainties in the absolute age calibration of the Valanginian time scale do not permit to demonstrate a precise synchronicity between environmental perturbations (dated by bio- and chemostratigraphies), and phases of intensified volcanic activity (dated by radiometric means). Moreover, other geochemical records of volcanic activity such as Os or Pb isotope records, are lacking for the Valanginian interval so far.

Since very recently, mercury (Hg) chemostratigraphy offers the possibly to evaluate the role of LIP activity during major palaeoenvironmental perturbations^25^. Volcanic and submarine hydrothermal emissions are considered as the major contributor of natural Hg in the ocean-atmosphere system^26^,^27^. Its enrichments are used to trace increased volcanic activity in both proximal and distal sites, because of its emission in the form of gaseous elemental mercury (Hg^0^), which is globally distributed due to a rather long atmospheric residence time (0.5–1 year)^28^. After oxidation to reactive Hg^2+^, Hg is deposited in continental and marine environments by precipitation^29^ (Fig. S1 in supplementary material). In sedimentary deposits, Hg is preferentially adsorbed onto organic matter, hydrous iron (Fe) oxides, and/or clay minerals^25^,^29^,^30^,^31^,^32^ (Fig. S1 in supplementary material). The Hg, total organic carbon (TOC) and/or phyllosilicate and iron contents are therefore generally correlated in modern and ancient sediments^29^,^30^,^31^,^32^. Consequently, the origin of sedimentary Hg anomalies is evaluated by discrmining between enrichments related to enhanced TOC, iron, and/or clay mineral contents and enrichments related to volcanic activity^29^. In fact, since the pioneering work of Sanei *et al*.^25^, anomalous enrichments in Hg contents observed in the geological records have been related to volcanic eruptions, such as the formation of the Siberian Traps leading to the end-Permian extinction event^31^,^33^, central Atlantic magmatic activity inducing end-Triassic mass extinction^34^, Karoo Ferrar LIP volcanism implied in the early Toarcian OAE^29^, and finally the build up of the Deccan Traps related to the K-T boundary extinction event^32^,^35^,^36^,^37^.

Here we investigate the distribution of Hg contents in four Valanginian reference sections located in pelagic and hemipelagic environments in the Central Tethyan Realm (Lombardian Basin, Breggia section), the northern Tethyan margin (Vocontian Basin, Orpierre and Angles sections), and the narrow seaway connecting the Tethyan and Boreal Oceans (Polish Basin, Wąwał core) (Fig. 1). The sedimentary succession of Breggia consists of a monotonous pelagic limestone succession. The Orpierre and Angles sections are composed of hemipelagic marl-limestone alternations, whereas the Wąwał core is comprised of monotonous sandy to silty clays. The reported Hg concentrations are normalized against TOC, iron (Fe) and phyllosilicate contents in order to discriminate variations due to local adsorptive processes from those due to increased volcanic activity^29^ (Figs 2 and 3). For the samples with less than <0.2 wt. % TOC, Hg/TOC ratios are not considered to reflect a realistic values^31^ and are consequently not shown here.

## Results

The selected sections have not been the subject of significant diagenesis, benefit from a robust temporal frameworks, and the evolution of the Valanginian Weissert episode is well defined by δ^13^C stratigraphy^6^,^16^,^38^,^39^,^40^; Fig. S2 in the supplementary material). All records show an enrichment in Hg concentrations at or near the onset of the Weissert episode, with maximal values of 70.5 ppb at Angles, 59.5 ppb at Orpierre, 69.9 ppb at Wąwał, and 17.0 ppb at Breggia (Figs 2 and 3). The Hg enrichments, situated in the *Campylotoxus* Zone, are abrupt and short-lived in the Orpierre and Wąwał sections. At Breggia and Angles the increases in Hg contents are equally well-defined and show a more gradual and longer-lived maximum. The Hg values are rather stable through the remainder of the Valanginian, with background values ranging between 20 and 27 ppb at Orpierre, Angles, and Wąwał; and between 5 and 8 ppb at Breggia.

## Discussion

The organic carbon record in the Vocontian Basin, expressed as TOC in weight percent, shows low values for the entire Valanginian interval, not exceeding 0.6 wt. %^11^,^16^. With regards to the Wąwał section, the TOC values are somewhat more elevated near the base and the top of the section to reach maxima of 1.24 and 1.44 w%, respectively^40^. However, the highest Hg contents are recorded during the Weissert episode where TOC values are below 0.7 wt% (Fig. 3). For all studied sections, the Hg/TOC ratios are rather well correlated with the overall Hg contents, and intervals of maxima in Hg/TOC ratios correspond to samples with relatively high Hg contents (Figs 2 and 3). At Breggia in the Lombardian Basin, at Breggia the TOC values remain constantly very low (<0.05 wt. %), and unlikely influenced Hg sequestration in the studied samples. Furthermore, normalizing Hg concentrations against Fe contents does not remove the trend observed in overall Hg contents in all sections, which suggest that hydrous iron oxides did not influence Hg sequestration^26^. We have also calculated Hg/phyllosilicate ratios for each section (Figs 2 and 3), and it appears that correlation coefficients between Hg and phyllosilicate are very low and that the observed Hg variations can not be explained by the presence of clay minerals alone (R^2^ = 0.01, 0.03, 0.15 and 0.13, at Orpierre, Angles, Breggia and Wąwał, respectively; Fig. S3 in the supplementary material). This implies that the Hg anomalies recorded in the studied sedimentary successions are not primarily controlled by organic-matter, hydrous iron oxides, and/or clay-mineral contents.

**Figure 1:**
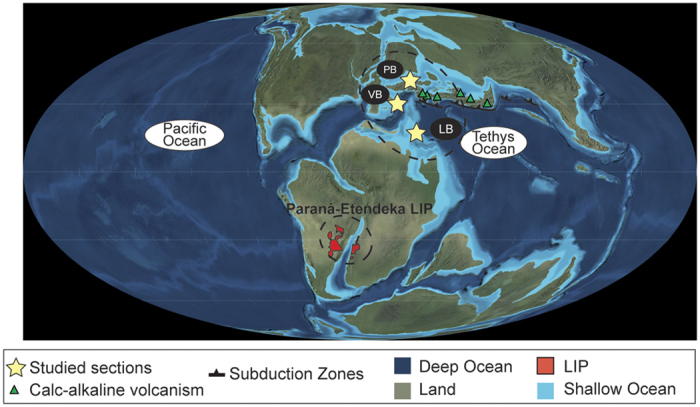
Study area: palaeogeographic map of the Early Cretaceous showing the location of the Paraná-Etendeka LIP, the Polish, Lombardian and the Vocontian Basins. (Figure modified from R. Blakey, http://cpgeosystems.com/euromaps.html).

In analogy to previous studies, in which anomalous enrichments in Hg were interpreted as indicative of an increase in volcanic activity^25^,^29^,^32^,^33^,^34^,^37^, we suggest that the Hg enrichments recorded in the European Valanginian sections are related to a rapid increase in atmospheric Hg concentrations associated with a massive release of volcanic Hg^0^ into the atmosphere just before the early/late Valanginian transition. A distal volcanic ash deposit occurs in the sediments from the Vocontian Basin during the *Campylotoxus* Zone, thus witnessing volcanic eruptions during the late early Valanginian^41^. The source of the volcanic ash deposit has been attributed to volcanic activity associated with the Tethyan subduction zone (ref. 41, Fig. 1), and implies that subduction-related volcanism was active near the onset of Weissert episode. Such process could have been a source of Hg input into the atmosphere. However, an alternative and more likely candidate for the main pulse in Hg contents is Paraná-Etendeka LIP activity, represented by the large intraplate magmatic province situated in southern South America and in southwest Africa (Fig. 1). A recent revision of the 40Ar/^39^Ar dates^23^ and U-Pb isotopic data on zircons^24^,^42^ points indeed to the short-lived character of this volcanic episode (not exceeding 1.2 Myr) with a main pulse at ~135 Ma^42^.

**Figure 2:**
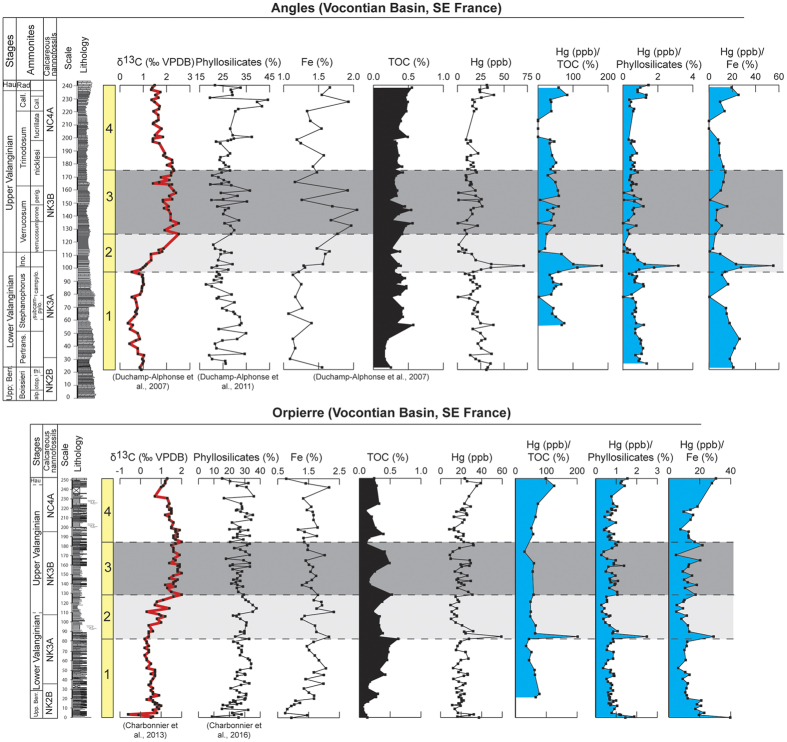
δ^13^C, phyllosilicate, iron (Fe), total organic carbon (TOC), Hg, Hg/TOC, Hg/phyllosilicate, and Hg/Fe from Angles and Orpierre sections. The biostratigraphic framework is based on ammonites and calcareous nannofossils^6^,^16^.

**Figure 3:**
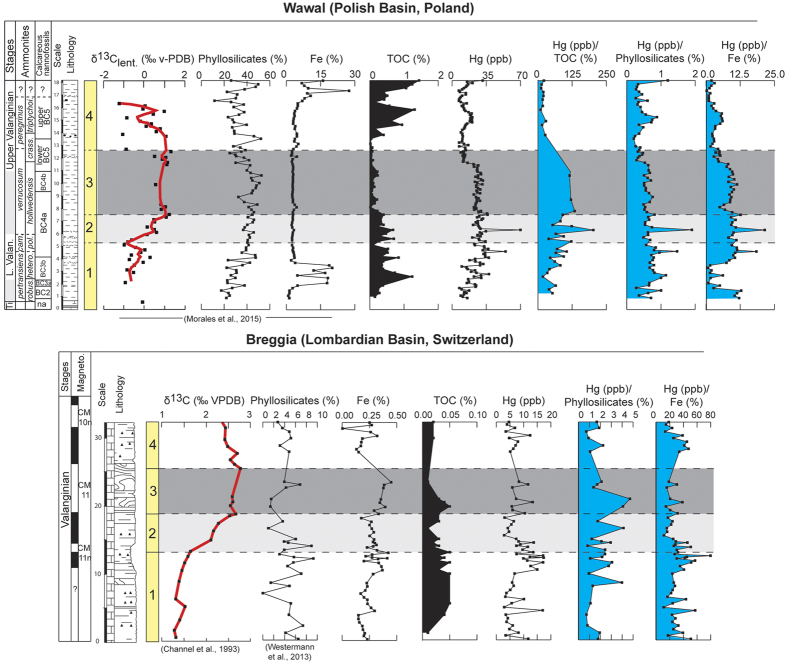
δ^13^C, phyllosilicate, iron (Fe), total organic carbon (TOC), Hg, Hg/TOC, Hg/phyllosilicate, and Hg/Fe from Wąwał and Breggia sections. The biostratigraphic framework is based on ammonites and magnetostratigraphy^38^,^40^.

The Hg anomalies documented herein are situated near the onset of the Weissert episode (Figs 2 and 3). They mark the starting point of major turnovers and crises recorded in marine and terrestrial ecosystems^3^,^11^,^12^,^13^,^14^,^15^,^16^,^43^,^44^. They allow us to directly establish a connection between an increase in volcanic outgassing and the Valanginian environmental and ecological changes. The initiation of volcanic activity and the hypothesized associated released of CO_2_ in the ocean-atmosphere system has been important enough to affect both the hydrological cycle and ocean chemistry. On one hand, it has accelerated the hydrological cycle, intensified silicate weathering, and fertilized the coastal environment, thereby affecting shallow-water ecosystems^3^,^14^. On the other hand, the excess amount of CO_2_ in surface waters have most probably been accompanied with lowered pH that could have hampered the development of several pelagic carbonate producers^45^. The demise of shallow-water carbonate platforms and the biocalcification crises in pelagic-environments coupled with a global increased in organic-matter burial and preservation, especially on continents, contributed to the positive shift in the δ^13^C records^46^. Volcanic activity was sufficiently important to impact the Valanginian biosphere and environment in a way that marine life severely suffered, whereas terrestrial life may have benefited from the prevailing warm and humid conditions, which lead to the development of widespread vegetation covers, favoring the evolution of herbivore life and eventually also providing a setting favorable to angiosperm evolution^8^.

## Methods

A total of 429 Hg analyses were achieved using a Zeeman R-915F (Lumex, St. Petersburg, Russia) high-frequency atomic absorption spectrometer at the University of Lausanne. Analyses are based on the direct thermal evaporation of Hg from solid samples. Measurements were systematically conducted on two aliquots. The accuracy was confirmed by the analysis of certified reference materials (GSD-11 standard, Chinese alluvium: 72.0 ppb)^47^ with a correlation coefficient of 0.99 and a standard residual deviation of 0.44. In complement, total organic carbon (TOC) at Orpierre and Breggia has been obtained by Rock-Eval^TM6^ analysis^48^ at the University of Lausanne. Approximately 50 to 70 mg of powdered sample material has been subjected to pyrolysis followed by complete oxidation of the residual sample^49^,^50^. Samples were calibrated using the IFP160000 standard with an instrumental precision of <2%.

## Additional Information

**How to cite this article**: Charbonnier, G. *et al*. Mercury enrichment indicates volcanic triggering of Valanginian environmental change. *Sci. Rep.* **7**, 40808; doi: 10.1038/srep40808 (2017).

**Publisher's note:** Springer Nature remains neutral with regard to jurisdictional claims in published maps and institutional affiliations.

## Supplementary Material

Supplementary Material

Supplmentary Data
