# Supplementary material for: Mercury enrichment indicates volcanic triggering of Valanginian environmental change
Source: Sci Rep. 2017 Jan 20;7:40808. doi: 10.1038/srep40808 (PMC5247756; doi:10.1038/srep40808)
Supplement: Supplementary Material [file srep40808-s1.pdf]

Mercury enrichment indicates volcanic triggering of Valanginian environmental change

Guillaume Charbonnier[1*], Chloé Morales[2], Stéphanie Duchamp-Alphonse[3], Stéphane Westermann[4], Thierry Adatte[1], Karl B. Föllmi[1]

1. Institute of Earth Sciences, Géopolis, University of Lausanne, CH-1015 Lausanne, Switzerland
2. Marine Palynology Group, Institute of Earth Sciences, University of Utrecht, Heidelberglaan 24, 3584 CS Utrecht, The Netherlands
3. Laboratoire GEOPS, Bâtiment 504, Université Paris Sud, UMR 8148, Orsay F91405, France
4. Cantonal agency for environmental protection, Rue des Creusets 5 CH-1950 Sion, Switzerland
* Corresponding author.
E-mail address: guillaume.charbonnier@unil.ch (G. Charbonnier)
Telephone number: 00 41 (0) 21 692 43 10

Supplementary Materials

Four sections situated in three different basins have been investigated in detail. The Lombardian Basin represents one of the major rift-related basins of the Mesozoic Tethys preserved in the southern Alps (*51*). The Valanginian deposits of the Breggia section consist of pelagic white grey limestones intercalated with chert nodules (*2*). The temporal framework of the section is assessed by calcareous nannofossil, magneto- and carbon-isotope stratigraphy (*2, 38, 39*). The Vocontian Basin is characterized by an hemipelagic sedimentation corresponding to palaeodepths of a few hundred meters, which is surrounded by three carbonate platforms (*52*). The Orpierre and Angles sections are composed of hemipelagic marl-limestone alternations of Valanginian age, which are well dated by ammonoïds, calcareous nannofossils and/or calpionellids, and carbon-isotope stratigraphy (*6*, *53-55*). The Angles section, in particular, has been used as a reference section to constrain the biostratigraphic framework of the Valanginian carbon cycle perturbation. The Polish Basin is located in a large transcontinental seaway ranging from 35 to 65°N, which connected the Artic Sea with the Tethys region (*56*). The Wąwał drill core is composed of Valanginian sandy and silty claystones dated by ammonites, calcareous nannofossils and $\delta^{13}C$ trends obtained from bulk-rock and monogeneric benthic foraminifera (*Lenticulina*) (*40*).

Precise chemostratigraphic correlations were established using the specific structure of the Valanginian $\delta^{13}C_{carb}$ curve (*3, 6, 16*). The early Valanginian (Unit 1) is marked by the lowest $\delta^{13}C_{carb}$ values (0.2-0.9‰). A shift towards more positive values is observed (amplitude 1.5-2‰) near the early-late Valanginian transition (Unit 2). An interval of relatively high $\delta^{13}C_{carb}$ values (between 1.4 and 2.0‰) persists during the late Valanginian (Unit 3), followed by a return towards pre-excursion values in the latest Valanginian (Unit 4) (Fig. S2). These $\delta^{13}C_{carb}$ records are well correlated with the carbon-isotope records from other parts of the Tethyan, Boreal, Atlantic or Pacific sites (*3, 5, 57, 58*).

Supplementary results and discussion

The relationship between Hg contents and phyllosilicate enrichments was investigated by direct correlation of the two data sets (Fig. S3). The lower correlation coefficient (R^2) between Hg concentrations and phyllosilicate contents measured in all sections (Orpierre: R^2=0.01, Angles: R^2=0.03, Breggia: R^2=0.15, Wąwał: R^2=0.13) suggests that the clay minerals and Hg concentrations are not correlated and therefore interdependent (Fig. S3).

Supplementary References

51. Weissert, H., & Bernoulli, D. A transform margin in the Mesozoic Tethys: evidence from the Swiss Alps. *Geol. Rundsch*. **74**, 665–679 (1985).

52. Masse, J.P. Valanginian-Early Aptian carbonate platforms from Provence, Southeastern France. In Simo, J.A.T., Scott R.W., Masse J.-P. (Eds), Cretaceous Carbonates Platforms. American Association of Petroleum Geologists Memoir, Tulsa, OK, United States. 363–374 (1993).

53. Allemann, F., & Remane, J. Les faunes de calpionelles du Berriasien supérieur/Valanginien. In Hypostratotype mésogéen de l'étage Valanginien (eds Busnardo, R., Thieuloy, J. P., Moullade, M.,), pp. 99–109 (CNRS, Paris) (1979).

54. Bulot, L.G., & Thieuloy, J.P. Les biohorizons du Valanginien du Sud-Est de la France: un outil fondamental pour les corrélations au sein de la Téthys occidentale. *Géologie Alpine, Mémoire H. S*. **20**, 15–41 (1994).

55. Gardin, S. et al. The Valanginian to Hauterivian hemipelagic successions of the Vocontian basin (SE France): new high resolution integrated biostratigraphical data. 6th International Cretaceous Symposium, Geozentrum. University of Vienna, Austria, p. 34 (2000).

56. Smith, A.G., Smith, D.G., & Funnell, B.M. Atlas of Mesozoic and Cenozoic Coastlines. Cambridge University Press, Cambridge (1994).

57. Bornemann, A., & Mutterlose, J. Calcareous nannofossil and $\delta^{13}C$ records from the Early Cretaceous of the western Atlantic ocean: evidence for enhanced fertilization across the Berriasian–Valanginian transition. *Palaios*. **23**, 821–832 (2008).

58. Kujau, A. et al. No evidence for anoxia during the Valanginian carbon isotope event-An organic-geochemical study from the Vocontian Basin, SE France. *Global and Planetary Change*. **92-93**, 92–104 (2012).

Supplementary figures

Fig. S1: The natural mercury cycle from volcanic emission to sedimentary deposition (figure modified from *29*).

Fig. S2: Correlation of the $\delta^{13}C$ records from Orpierre (*6*), Angles (*16*), Wąwał (*40*), and Breggia (*38*).

Fig. S3: Hg (ppb) versus phyllosilicate (%) diagram of the studied Valanginian successions.

Fig. S1

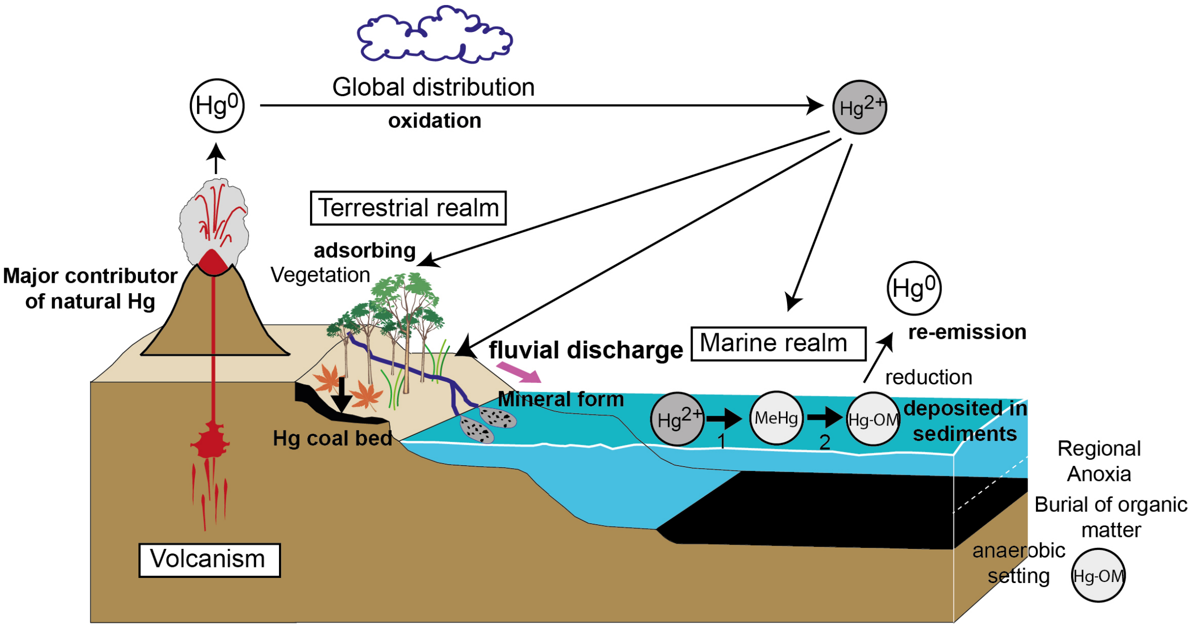

1: Biotic or abiotic processes from a mercury sulphide complex (HgS) or methylmercury (MeHg)
2: Bioacumulation of lipophilic MeHg forms an organo-mercury complex (HgOM).
HgOM is transferred into sediments and accumulated .

Fig. S2

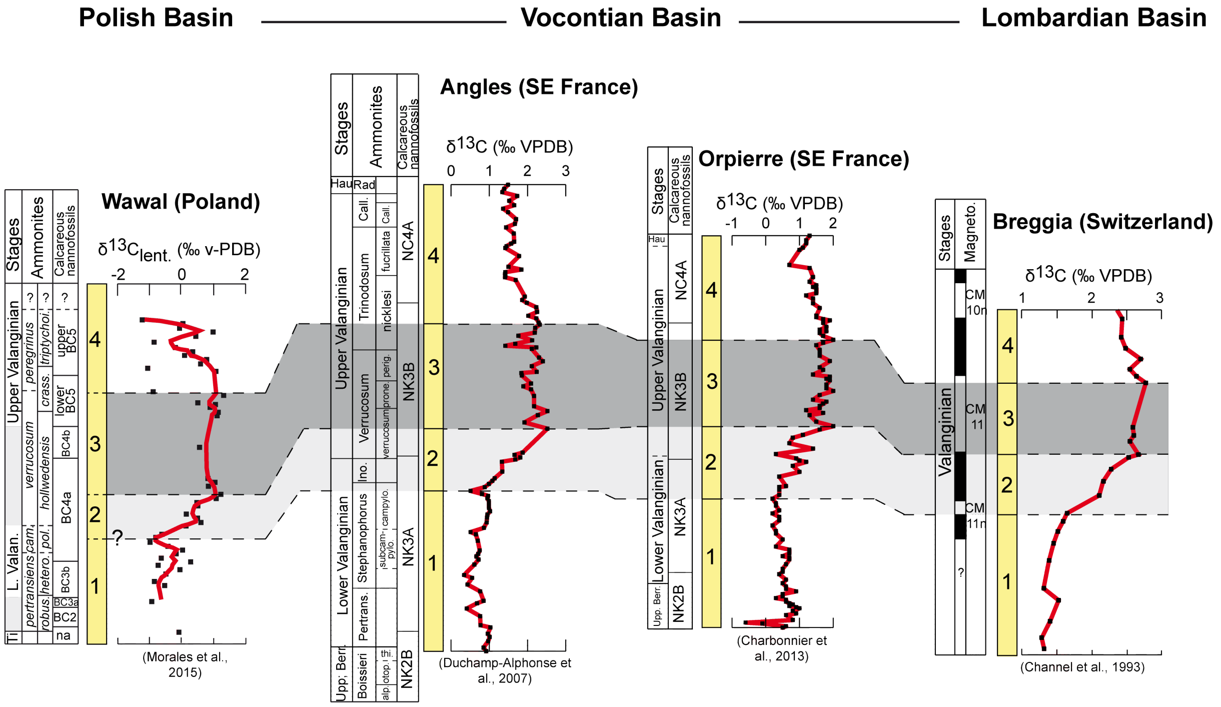

Polish Basin
Vocontian Basin
Lombardian Basin
Wawal (Poland)
δ13Clent. (‰ v-PDB)
Stages
Ammonites
Calcareous nannofossils
Upper Valanginian
L. Valan.
(Morales et al., 2015)
Angles (SE France)
δ13C (‰ VPDB)
Upper Valanginian
Lower Valanginian
Verrucosum
Trinodosum
Stephanophorus
Pertrans.
Boissieri
NC4A
NK3B
NK3A
NK2B
(Duchamp-Alphonse et al., 2007)
Orpierre (SE France)
δ13C (‰ VPDB)
(Charbonnier et al., 2013)
Breggia (Switzerland)
Magneto.
Valanginian
δ13C (‰ VPDB)
(Channel et al., 1993)

Fig. S3

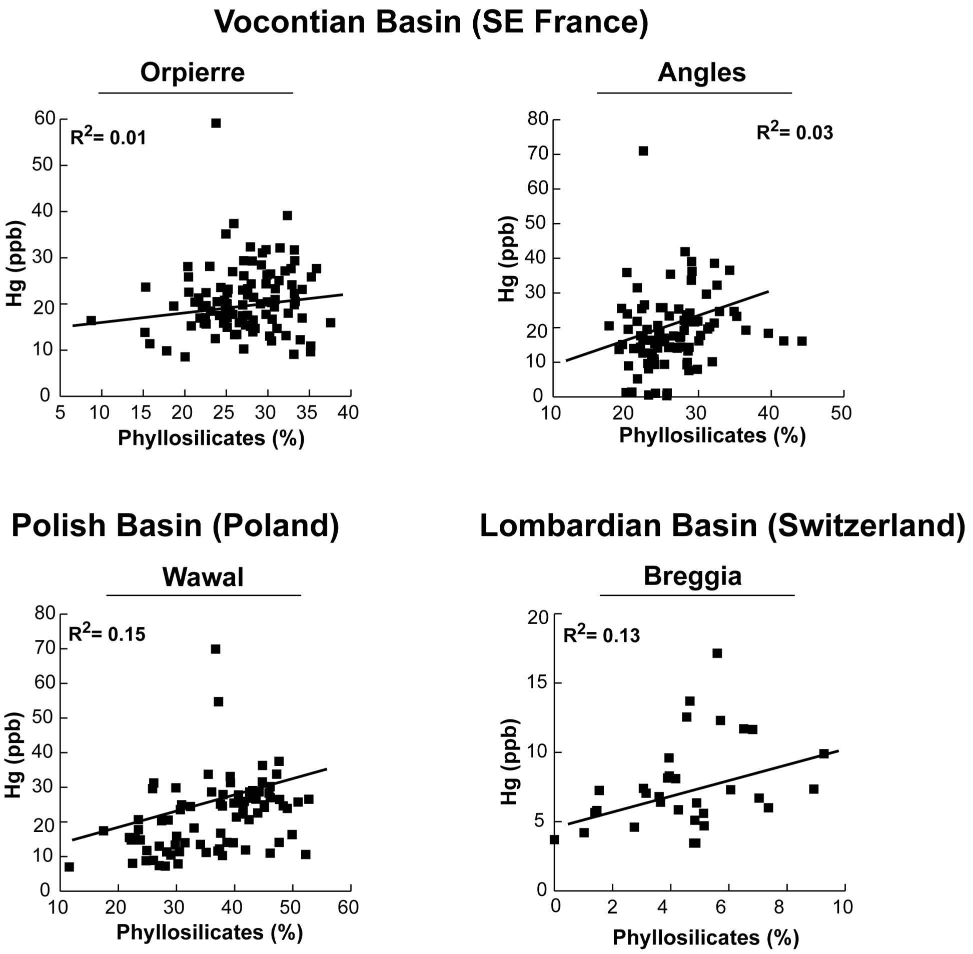

Vocontian Basin (SE France)
Orpierre
Angles
Polish Basin (Poland)
Lombardian Basin (Switzerland)
Wawal
Breggia
R^2= 0.01
R^2= 0.03
R^2= 0.15
R^2= 0.13
Hg (ppb)
Phyllosilicates (%)
